# Identification and Functional Analysis of the Pheromone Response Factor Gene of *Sporisorium scitamineum*

**DOI:** 10.3389/fmicb.2019.02115

**Published:** 2019-09-10

**Authors:** Guining Zhu, Yizhen Deng, Enping Cai, Meixin Yan, Guobing Cui, Zhiqiang Wang, Chengwu Zou, Bin Zhang, Pinggen Xi, Changqing Chang, Baoshan Chen, Zide Jiang

**Affiliations:** ^1^Guangdong Province Key Laboratory of Microbial Signals and Disease Control, State Key Laboratory for Conservation and Utilization of Subtropical Agro-Bioresources, Department of Plant Pathology, South China Agricultural University, Guangzhou, China; ^2^Guangxi Key Laboratory of Biology for Crop Diseases and Insect Pests, Plant Protection Research Institute, Guangxi Academy of Agricultural Sciences, Nanning, China; ^3^State Key Laboratory for Conservation and Utilization of Subtropical Agro-Bioresources, College of Life Science and Technology, Guangxi University, Nanning, China

**Keywords:** *Sporisorium scitamineum*, pheromone response factor, mating, pathogenicity, fungi

## Abstract

The sugarcane smut fungus *Sporisorium scitamineum* is bipolar and produces sporidia of two different mating types. During infection, haploid cells of opposite mating types can fuse to form dikaryotic hyphae that can colonize plant tissue. Mating and filamentation are therefore essential for *S. scitamineum* pathogenesis. In this study, we obtained one T-DNA insertion mutant disrupted in the gene encoding the pheromone response factor (Prf1), hereinafter named *SsPRF1*, of *S. scitamineum*, via *Agrobacterium tumefaciens*-mediated transformation (ATMT) mutagenesis. Targeted deletion of *SsPRF1* resulted in mutants with phenotypes similar to the T-DNA insertion mutant, including failure to mate with a compatible wild-type partner strain and being non-pathogenic on its host sugarcane. qRT-PCR analyses showed that *SsPRF1* was essential for the transcription of pheromone-responsive mating type genes of the *a1* locus. These results show that *SsPRF1* is involved in mating and pathogenicity and plays a key role in pheromone signaling and filamentous growth in *S. scitamineum*.

## Introduction

Sugarcane smut caused by *Sporisorium scitamineum* is a devastating disease in sugarcane worldwide. Plants infected with the pathogen are severely stunted with thin stalks, producing no millable canes. The most recognizable characteristic of this disease is a black or gray growth from the top of plant cane that is referred to as a “smut whip” that is composed of a central core of pithy plant tissue surrounded by the fruiting structures of the fungus and the brown to black teliospores (Croft and Braithwaite, 2006; Sundar et al., 2012). *S. scitamineum* is bipolar and produces sporidia of two opposite mating types, *MAT-1* and *MAT-2* (Yan et al., 2016b). Sporidia of different mating types can fuse to form pathogenic dikaryotic hyphae to infect sugarcane buds and the hyphae grow within the meristematic tissue, eventually producing whip-like fruiting structure and teliospores in the infected plants. The diploid teliospores germinate and undergo meiosis to yield haploid sporidia, which need to mate again to infect the plant and to initiate a next round of infection (Albert and Schenck, 1996; Croft and Braithwaite, 2006; Yan et al., 2016b). Thus, mating plays a central role in the life cycle of smut pathogens, as it initiates parasitism by a morphological and physiological transition from saprotrophic yeast cells to pathogenic filaments (Hartmann et al., 1996; Bakkeren et al., 2008; Kellner et al., 2011).

The conserved MAPK and cAMP/PKA signaling pathways regulate important aspects of fungal virulence in various pathogenic fungi such as *Magnaporthe oryzae* (Marroquin-Guzman and Wilson, 2015), *U. maydis* (Mayorga and Gold, 1999), and *Setosphaeria turcica* (Li et al., 2016) etc. The life cycle of *S. scitamineum* is similar to that of the well-studied model fungus *Ustilago maydis* that causes corn smut disease. In *U. maydis*, mating is regulated by two loci, *a* and *b*. The biallelic *a* locus (*a1* and *a2*) encodes pheromone precursors *mfa1* and *mfa2*, respectively, and receptors *pra1* and *pra2*, respectively. The pheromone-receptor system is required for recognition and fusion of haploid sporidia, while the multiallelic *b* locus encodes *bE* and *bW*, subunits of a heterodimeric transcription factor that regulates filamentation, dikaryon maintenance and pathogenicity. The expression of the genes in the *a* and *b* loci is induced upon pheromone stimulation (Hartmann et al., 1996). Basal as well as pheromone-induced transcription of these mating type genes is regulated by the pheromone responsive factor Prf1, which binds specifically to the pheromone response elements (PREs) located in the vicinity of all pheromone-inducible genes at the *a* and *b* loci (Hartmann et al., 1996; Urban et al., 1996). The activity of Prf1 is, in turn, regulated by the cyclic-AMP (cAMP) signaling pathway and the mitogen-activated protein (MAP) kinase module (Regenfelder et al., 1997; Krüger et al., 1998; Müller et al., 1999, 2003; Andrews et al., 2000; Kaffarnik et al., 2003). Prf1 is also regulated by other regulators such as *rop1*, *hap2* and *med1* (Brefort et al., 2005; Zarnack et al., 2008; Mendoza-Mendoza et al., 2009; Chacko and Gold, 2012).

In recent years, the whole genome sequences of *S. scitamineum* strains from China, Brazil, Australia and South Africa have been reported (Que et al., 2014; Taniguti et al., 2015; Dutheil et al., 2016). The availability of genome sequences facilitates the investigation of the pathogenicity mechanism of *S. scitamineum*. To understand the molecular basis of sexual mating and pathogenic development in *S. scitamineum*, we developed an efficient *Agrobacterium tumefaciens*-mediated transformation (ATMT) system (Sun et al., 2014) and identified the *b*-locus as essential for sexual mating and filamentous growth in *S. scitamineum* (Yan et al., 2016b). During the screening of an ATMT transformant library, we found a T-DNA insertion mutant, 248E3, that was unable to mate or form filamentous hyphae. The disrupted gene was identified as an ortholog of the *U. maydis PRF1* gene, designated as *SsPRF1*. Functional characterization of *SsPRF1* revealed that it functions as a pheromone response regulator, plays an essential role in the regulation of *a* locus gene expression, and is required for *S. scitamineum* pathogenicity.

## Materials and Methods

### Strains and Growth Conditions

The compatible haploid strains JG35 (*MAT-2*) and JG36 (*MAT-1*) of *S. scitamineum* were isolated from germinated teliospores collected from the sugarcane smut in Guangxi, China (Lu et al., 2017). Cultures were grown in YePS broth medium (1.0% yeast extract, 2.0% peptone, 2.0% sucrose) on a rotary shaker at 200 rpm at 28°C or on solid YePS agar at 28°C.

### Plant Materials

Sugarcane seedlings of the highly susceptible variety, ROC22, were cultivated in B. Chen’s experimental field in Guangxi University, and used for pathogenicity assay.

### Molecular Manipulations

Plasmid DNA was isolated with the SanPrep plasmid mini kit (Sangon, B518191) and *S. scitamineum* genomic DNA was extracted using the method described previously (Yan et al., 2016b). For Southern blot analysis, DNA samples (3–5 μg) digested with appropriate restriction enzymes were separated by electrophoresis and blotted to Hybond N^+^ membrane. Standard hybridization and detection protocols were performed using the method of DIG DNA labeling and detection kit (Roche, 11093657910). Total RNA was isolated with TRNzol-A^+^ (Tiangen, DP421) and first-strand cDNA was synthesized using the Revert AidFirst Strand cDNA Synthesis Kit (Thermo Fisher Scientific, K1621).

### ATMT Mutagenesis and Identification of T-DNA Insertion Site

*Agrobacterium tumefaciens*-mediated transformation (ATMT) was used to generate mutants of *S. scitamineum* haploid strains JG35 and JG36 using the *A. tumefaciens strain* AGL1 (Sun et al., 2014). The transformation procedure was adapted and modified from the methods developed for *U. maydis* (Ji et al., 2010). T-DNA left-border flanking sequence was amplified by high-efficiency TAIL-PCR (hiTAIL-PCR; Bölker et al., 1992; Liu and Chen, 2007), using genomic DNA of T-DNA insertion mutants as templates. Two rounds of amplifications were performed. Specific primers (LB1, LB2, and LB3) in combination with arbitrary degenerate primer (LAD1-4) and AC1 were used. The primer pair LB1/LAD1-4 was used in the pre-amplification step, while the primer pairs AC1/LB2 and AC1/LB3 were used in the primary and secondary-amplification, respectively. Primer sequences are listed in Table 1. The primary TAIL PCR product of about 850 bp was purified using the PCR-Clean kit (Sangon, SK8142), cloned into pMD 18-T Vector (TaKaRa, 6011), and sequenced using M13 forward or reverse primer.

**TABLE 1:** Primers used in this study.

**Name**	**Sequence**
LAD1-4	5′ACGATGGACTCCAGAGCGGCCGC(G/C/T)(G/A/T)N(G/C/T) NNNCGGT
AC1	5′ACGATGGACTCCAGAG
LB1	5′TGACGGCAATTTCGATGATGCAGC
LB2	5′GGACCGATGGCTGTGTAGAAGTAC
LB3	5′CGATCGACAAGCTCGAGTTTCTCC
M13-R	5′TCACACAGGAAACAGCTATGACC
M13-F	5′CAGGGTTTTCCCAGTCACGAC
hpH1-F	5′GCAAGACCTGCCTGAAACCG
hpH1-R	5′GGTCAAGACCAATGCGGAGC
*prf1-*P1	5′AAAGTTTAAACTGCTCTGTGCCACGCCTTGA
*prf1-*P2	5′AAACTGCAGCTGACTACAGACGATGTTGGTGGT
*prf1-*P3	5′AAAACTAGTCTGTAGGATGCAATGTATAGGC
*prf1-*P4	5′AAAGGATCCTGGAAGGTTGGTGCGAGA
*prf1-*P5	5′CACTAGTGGTACAACACCGAC
*prf1-*P6	5′GCAAACCTGCCTATCAGCAAG
*prf1-*P7	5′GTGTGAGTAGTTCCCAGATAAGGG
*prf1-*P8	5′AGGTGTGAAAACGATGCGATG
248E3-F	5′ATGCGAGACCAAGCTACCAC
248E3-TR	5′CGTAATGGGCACGATCTTCGG
prf1-2R	5′AAAGGATCCCTACGTGTAGTGTCCATTCCAA
Nat-F	5′CACTCTTGACGACACGGCTT
Nat-R	5′GCATGCTCATGTAGAGCGC
248E3-qF	5′GTCGACCTCTTTCACGGATG
248E3-qR	5′CTCGCTTGGGAAAGGAGATG
pra1-qF	5′CGAGTGTTGCCATGTTGGAGAGT
pra1-qR	5′TTGTAGCCTTGACGAACTTCCTGAC
mfa1-qF	5′TCTTTACCCAGACCGCCCAGAC
mfa1-qR	5′GGTGCAGCTAGAGTAGCCAAGG
bE1-qF	5′TGAAAGTTCTCATGCAAGCC
bE1-qR	5′TGAGAGGTCGATTGAGGTTG
bW1-qF	5′CACGTTGGATCTCGCTCGGT
bW1-qR	5′TCGGAAGGGAGGACGCAAAC
18S-qF	5′GACACCTCAACTCAGCGACACAA
18S-qR	5′TGCCCTTGCCATAGTCCCAAATG
248E3-F3	5′GAGAAGGCTACGAGCCAGTT
248E3-R	5′GCAAATGTAGTGCAATGACGC
*mfa1-*P1	5′AAAGTTTAAACGCTGTGTTGTTGATTGAGAGTGG
*mfa1-*P2	5′AAAAAGCTTGTTGGTGTTCGCAAGAACGA
*mfa1-*P3	5′AAAACTAGTTGCTGACGAATGTGTCCTTC
*mfa1-*P4	5′AAAGGATCCCGATTGTGATAGTGTGAGAGAGAG
*mfa1-*P5	5′TCAAGCGATAATGCAGCCAGTC
*mfa1-*P8	5′GGATGGCTTCATCGTGTGTACTGT
*pra1-*P1	5′AAAGTTTAAACAGGTAGACGCATCCGATCCA
*pra1-*P2	5′AAACTGCAGGACAAGCCGAAGTGGTGATG
*pra1-*P3	5′AAAACTAGTACATGGCTTCGTTGTCGAGT
*pra1-*P4	5′AAAGGATCCAGCTGTGCTCTTCTTGCTCTT
*pra1-*P5	5′GAGAACCTTGAGCAAGTGCTCG
*pra1-*P8	5′CGAGAAATGGTGTCACAAGACGAT
*pra1-*F	5′ATGCTTGACCACGTAACACCT
*pra1-*R	5′CGCAGATATCGAGGTAGTCACA

### Plasmid Construction for *SsPRF1* Deletion and Complementation

Binary vectors pEX1-GAP-eGFP, pNGR1, and pEX2, all derived from the binary T-DNA vector pPZP200 (Hajdukiewicz et al., 1994) and with T-DNA, were kindly provided by Dr. Lianghui Ji from National University of Singapore (Ji et al., 2010). *SsPRF1* deletion construct was made according to protocol previously described (Ji et al., 2010). Briefly, a 1577 bp upstream fragment (HS1) and a 841 bp downstream fragment (HS2) of the *SsPRF1* gene were amplified with primer pairs *prf1*-P1/*prf1*-P2 and *prf1*-P3*/prf1*-P4, respectively (Table 1) using JG36 genomic DNA as template. The HS2 fragment was digested with *Spe*I and *Bam*HI and ligated into pEX2 to produce pEX2-HS2. The HS1 fragment was digested with *Pme*I and *Pst*I and then ligated into pEX2-HS2 at the corresponding sites to yield the gene knock out construct pEX2-Δ*Prf1*. The recombinant plasmid pEX2-Δ*Prf1* was introduced into *Agrobacterium* strain AGL1 and used for transformation of JG35 and JG36 sporidia (Sun et al., 2014). Transformants were screened by PCR using primer pairs *prf1-*P5/*prf1-*P6, *prf1-*P7/*prf1-*P8, and 248E3-F/248E3-TR (Table 1), respectively. Locations of the primers were illustrated in Figure 3B. Gene knockout and absence of ectopic copy were further confirmed by Southern blot analysis as described.

For functional complementation assays, the primer pairs prf1-P1/prf1-2R (Table 1) were used to amplify a 4627 bp fragment carrying the entire *SsPRF1* gene from JG36 genomic DNA. The PCR product was digested with *Pme*I and *Bam*HI and cloned into the Nourseothricin-resistance vector pNGR1 at the corresponding sites to yield the complementary plasmid pCPrf1. The plasmid pCPrf1 was introduced into *Agrobacterium* strain AGL1 and used for transformation of the T-DNA insertion mutant 248E3.

### Mating Assays

Assay for mating was performed as previously described (Yan et al., 2016b). The *S. scitamineum* transformants were picked from the selection plates and inoculated in YePS medium (supplemented with 300 μg/ml Cefotaxime) for 2 days at 28°C on a rotary shaker. The 2 days cultures of wild-type opposite mating type strains (JG35, JG36, or mutants) were mixed with equal amounts of cells and a small drop was spotted on YePS-agar plate and incubated at 28°C for observation of colony morphology.

### Pathogenicity Assay

Pathogenicity assay was performed using the method described by Yan et al. (2016b). Fungal strains or mutants were grown in YePS broth for 2 days in a shaking incubator at 28°C and then collected by centrifugation to remove the medium. Fungal cells were resuspended in sterilized double-distilled water adjusted to 1 × 10^6^ cells/ml before mixing with the opposite mating type for inoculation. Sugarcane seedlings of the highly susceptible variety, ROC22, at 5–6 leaf stage grown in greenhouse were inoculated by injecting the stem near the growing point with approximately 200 μl of the mixed culture per plant. The control plants were injected with sterilized double-distilled water. Three biological repeats were applied in the inoculation and each repeat contained 5 plants grown in a pot of 30 cm in diameter. Pathogenicity was examined and documented till 120 days post-inoculation when the characteristic symptoms of a “smut whip” could be fully observed on the sugarcane plants inoculated with the positive control of JG36/JG35.

For fungal biomass assessment in the infected sugarcane seedlings, the sugarcane stem tissue infected by *S. scitamineum* sporidia (compatible mating types mixing) was collected at 72 hpi. Quantification of relative fungal biomass in infected sugarcane stem tissue was performed using the fungal *ACTIN* gene as reference, and sugarcane glyceraldehyde dehydrogenase (*GAPDH*) gene as reference for normalization, following the established protocol (Sun et al., 2019).

### Transcriptional Profiling

Strains or mutants pre-grown in YePS broth for 2 days at 28°C with shaking were diluted to 0.1 OD_600_ with YePS. Equal amounts of the diluted cells of JG35 were mixed with those of JG36, JG36Δ*prf1*, and 248E3, respectively and 50 ml of the mixtures or the haploid strains were incubated for 2 days at 28°C on a rotary shaker. Cultures were harvested by centrifugation and total RNA was isolated from haploid strains or mixture of strains. Two μg of purified total RNA of each sample was used as a template for first-strand cDNA synthesis using the RevertAid^TM^ First Strand cDNA Synthesis Kit (Thermo Fisher Scientific, K1621). For real-time quantitative polymerase chain reaction (qRT-PCR) assay, the cDNA samples were diluted 20-fold with ddH_2_O and used as qRT-PCR templates. The specific primer pairs of 248E3-qF/248E3-qR, mfa1-qF/mfa1-qR, pra1-qF/pra1-qR, and 18S-qF/18S-qR were used to amplify the target genes, *SsPRF1*, *SsMFA1*, *SsPRA1*, and *18S rRNA*, respectively. *18S rRNA* was used as the reference gene for expression normalization of the target genes and wild-type strain JG36 was used as the calibrator. qRT-PCR was performed in a LightCycler^®^ 480 Real-time PCR system (Roche). Amplification reaction contained 10 μl of 2 × SYBR Green I Master Mix (Roche, 4707516001), 1.0 μl of each primer (10 μM), 1.0 μl of template cDNA and nuclease-free water was added to a final volume of 20 μl. The cycling parameters were: 95°C for 10 min, followed by 50 cycles of 95°C for 10 s and 60°C for 60 s. Then, the PCR products were heated to 95°C with 4.4°C/s, cooled to 65°C with 2.2°C/s, and heated to 97°C with 0.11°C/s. Negative controls were reactions without template or transcriptase and were included in each experiment set. qRT-PCR reactions were performed for three technical replicates and three biological replicates for each sample. For comparison of gene expression, the average threshold cycle (*C*t) values for target genes and the house-keeping gene *18s rRNA* were first calculated and then relative quantification was calculated using the comparative 2^–Δ^ ^ΔCt^ method (Livak and Schmittgen, 2001). Data were normalized with endogenous *18s rRNA* level from the same samples.

## Results

### Characterization of the T-DNA Insertion Mutant 248E3

**FIGURE 1:**
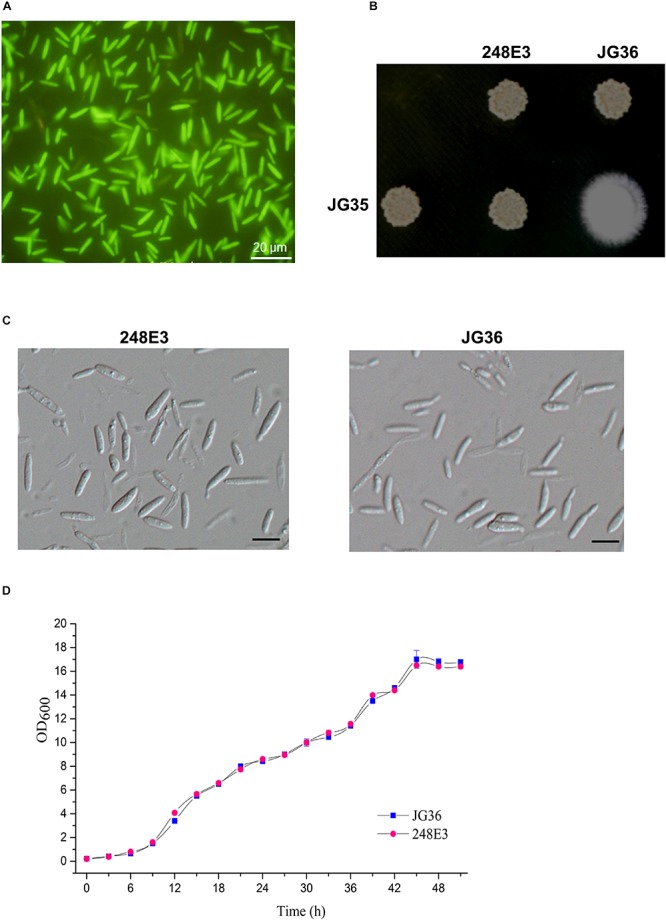
Characterization of T-DNA insertion mutant 248E3. **(A)** Fluorescence imaging of the mutant haploid sporidia. Photographs were taken at day 3 after inoculation onto the YePS plates. Scale bar = 20 μm. **(B)** Mating behavior of mutant 248E3. The mutant was co-spotted with compatible partner JG35 on YePS plate and incubated at 28°C for 3 days. **(C)** Differential interference contrast (DIC) images of sporidia: mutant 248E3 and JG36 (wild type). Scale bar = 10 μm. **(D)** Growth curves of mutant 248E3 and JG36 in YePS liquid medium. Cultures were kept at 28°C in a rotary incubator at 200 rpm. The initial OD_600_ value was 0.2 and was measured once every 3 h. Data give averages ± SE of three technical replicates conducted at the same time.

To identify genes essential for *S. scitamineum* mating/filamentation, we constructed an ATMT mutant library with two compatible mating-type strains of *S. scitamineum*, JG36 (*MAT-1*), and JG35 (*MAT-2*). A total of 25056 transformants with hygromycin resistance marker were obtained, in which 15873 transformants were derived from JG36 and 9183 from JG35. All the T-DNA insertion mutants exhibited bright green fluorescence under epifluorescent microscope (Figure 1A), confirming the presence of a T-DNA cassette carrying an eGFP in the *S. scitamineum* genome (Supplementary Figure S1A).

We then screened these transformants for mating defects. Mixing of the wild-type strain JG36 (*MAT-1*) with the compatible wild-type mating partner JG35 (*MAT-2*) gave rise to a fluffy colony, producing visible thin and white aerial filaments at 1–2 days post-spotting on YePS-agar plate (Figure 1B), indicating successful mating and formation of dikaryotic hyphae. Six isolates showing glossy appearance of mating mixture, indicative of mating defects, were selected and subjected to a second round of mating test. Among these was the mutant 248E3 from JG36 background, which was unable to produce dikaryotic hyphae when co-spotted with JG35 (Figure 1B), confirming that it was defective in mating. Apart from mating defect, 248E3 appeared indistinguishable from the wild type strain JG36, in colony and basidiospore (sporidium) morphology (Figures 1B,C) and the growth rate in YePS liquid medium (Figure 1D; *p* > 0.05).

### Identification of the *S. scitamineum PRF1* Homolog

**FIGURE 2:**
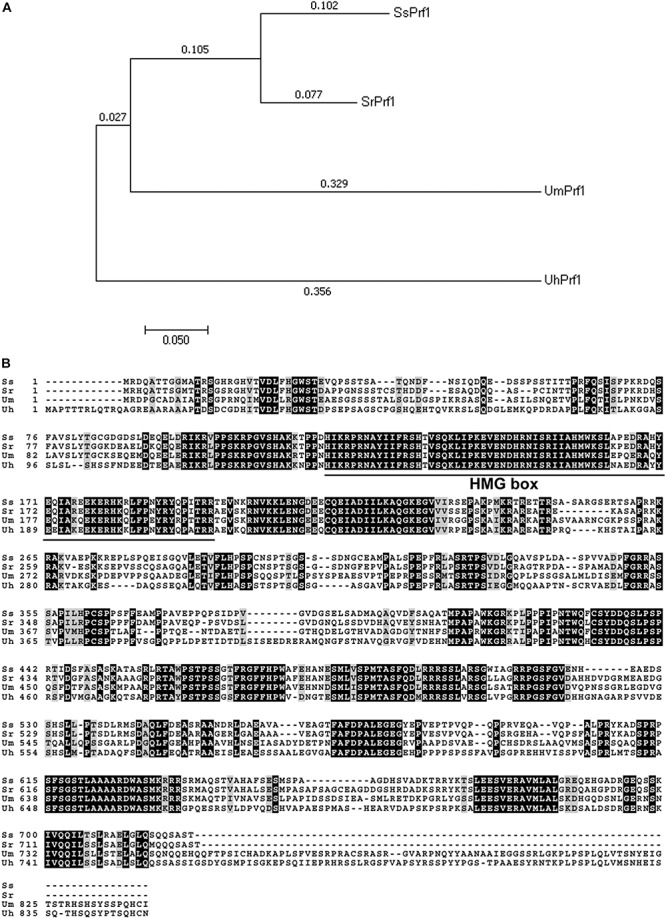
Analysis of fungal *PRF1* orthologs. **(A)** Phylogenetic analysis of four Prf1 orthologs in fungi was conducted in MEGA7 (Kumar et al., 2016) using the Neighbor-Joining method (Saitou and Nei, 1987). The optimal tree with the sum of branch length = 0.99597394 is shown. The percentage of replicate trees in which the associated taxa clustered together in the bootstrap test (1000 replicates) is shown next to the branches (Felsenstein, 1985). The tree is drawn to scale, with branch lengths in the same units as those of the evolutionary distances used to infer the phylogenetic tree. The evolutionary distances were computed using the Jones-Taylor-Thornton (JTT) matrix-based method (Jones et al., 1992) and are in the units of the number of amino acid substitutions per site. SrPrf1: CBQ73103.1; UmPfr1: AAC32736.1; UhPrf1: CCF52951.1. **(B)** Sequence alignments of the above mentioned fungual SsPrf1 proteins was performed using Boxshade server (https://embnet.vital-it.ch/software/BOX_form.html), with the aligned sequence generated by Clustal Omega (www.ebi.ac.uk/Tools/services/web/toolresult.ebi? jobId=clustalo-I20180725-032536-0068-98009260-p2m&showColors=true&tool=clustalo). A conserved domain (MATA_HMG-box, class I member of the HMG-box superfamily of DNA-binding proteins) is underlined.

Southern blotting analysis showed that mutant 248E3 contains a single copy of T-DNA inserted into its chromosome (Supplementary Figure S1B). By high-efficiency TAIL-PCR with the primers LAD1-4, AC1 paired with LB1, LB2 and LB3 (Table 1 and Supplementary Figure S1C), a fragment of about 850 bp was obtained using 248E3 genomic DNA as template (Supplementary Figure S1D). Sequence analysis showed that this fragment contained a 348 bp stretch of the genome sequence. Using this 348 bp fragment to screen the genome sequence of *S. scitamineum* in the NCBI database, we identified a putative gene (ID: *SSCI14340.1*), in which the T-DNA was inserted inside its coding sequence at coordinate 1033 bp from the translation start codon (Supplementary Figure S1C). The open reading frame (ORF) of *SSCI14340.1* was 2166 bp, encoding a predicted polypeptide of 722 amino acids (GenBank: CDW96669.1). This protein shows 78% identity to the probable pheromone response factor Prf1 of *S. reilianum* (GenBank: CBQ73103.1), 57% to Prf1 of *U. maydis* (AAC32736.1) and 56% to a protein related to pheromone response factor Prf1 of *U. hordei* (CCF52951.1), respectively (Figure 2A). All these fungal Prf1 orthologs contain a highly conserved domain of HMG box superfamily at the N-terminus (Figure 2B). Therefore we named this gene *SsPRF1*. By sequencing the RT-PCR products using total RNA from JG35 (*MAT-2*), we found that *SsPRF1* was also present in the strain with the opposite mating type. Southern blotting analysis confirmed that this gene was present in both JG35 and JG36 with a single copy in their genomes (Supplementary Figure S1B).

### *SsPRF1* Is Essential for Sexual Mating in *S. scitamineu*m

**FIGURE 3:**
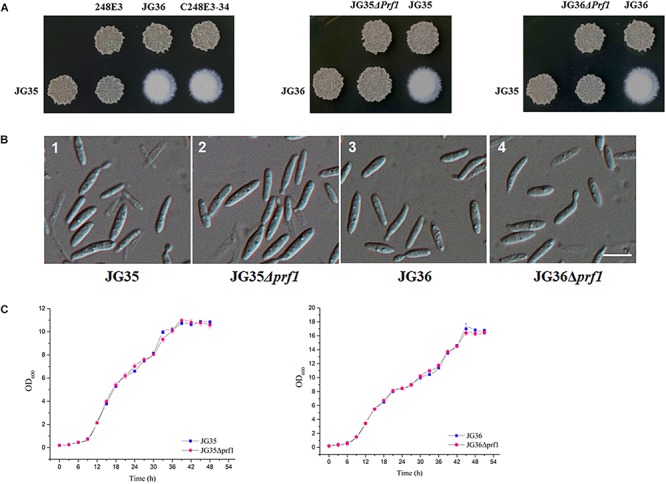
Characterization of *SsPRF1* deletion or complementation strains. **(A)** Mating assays of mutants. Cultures of single wild-type strains and *SsPRF1* mutants indicated on the top and on the left side of the photographs were spotted alone or in combination on YePS plates and incubated at 28ş C for 3 days. Colonies on the first line on the photographs are wild-type strains or mutants spotted alone, while colonies on the second line are wild-type strains JG35 or JG36 spotted alone or in combinations with strains indicated at the same columns. Positive mating reaction is indicated by the white fluffy colony morphology. Combinations of mutant strains show that T-DNA disrupted *SsPRF1* mutant 248E3 and *SsPRF1* null mutants JG35Δ*prf1* and JG36Δ*prf1* failed to form white and fluffy colonies and complementation strain C248E3-34 restored the wild type phenotype of mating to form fuzzy colony when tested with compatible, wild-type mating partners. **(B)** DIC images of sporidia of *SsPRF1* deletion mutants and wild-type strains. 1, 2, 3, 4 represent JG35, JG35Δprf1, JG36, JG36Δprf1, respectively; Scale bar represents 10 μm. **(C)** Growth curve of JG35Δ*prf1* and JG36Δ*prf1* mutants in comparison to the wild-type strains. Sporidia of each strain were cultured in YePS liquid medium, at 28°C with shaking at 200 rpm. Initial culture was adjusted to OD_600_ = 0.2, and the OD_600_ values were measured every 3 h. Mean ± S.E. was derived from three independent repeats.

To further demonstrate the functionality of *SsPRF1*, a 4.6 kb fragment containing the entire *SsPRF1* gene of *S. scitamineum MAT-1* (JG36) strain was cloned into the vector pNGR1 to result in the complementation plasmid pCPrf1. This plasmid was introduced into the T-DNA insertion mutant 248E3 to generate the complementation transformant C248E3-34. Sporidia of the complementation strain C248E3-34 were able to form white fluffy colonies when co-spotted with a compatible wild-type mating partner JG35 (*MAT-2*), similar to the sexual mating between the two wild-type mating-type strains (Figure 3A).

We further generated the *SsPRF1* null mutants in both JG35 and JG36 background by homologous recombination (Supplementary Figure S2A). After antibiotic selection and two rounds of successive screening by mating tests, the *SsPRF1* null mutants JG35Δ*prf1* from JG35 and JG36Δ*prf1* from JG36 were verified by PCR amplification (Supplementary Figures S2B,C) and confirmed by Southern blotting (Supplementary Figure S2D). In agreement with the phenotype of the T-DNA insertion mutant 248E3, the morphology of these *SsPRF1* deletion mutants appeared indistinguishable from the wild-type strain (Figure 3B), except that they both failed to form fluffy colonies when co-spotted with compatible wild-type mating partners (Figure 3A). The growth rates of the *ssprf1*D mutants in YePS liquid medium were also comparable to that in their wild-type strains (Figure 3C; *p* > 0.05).

### *SsPRF1* Is Required for *S. scitamineum* Pathogenicity

**FIGURE 4:**
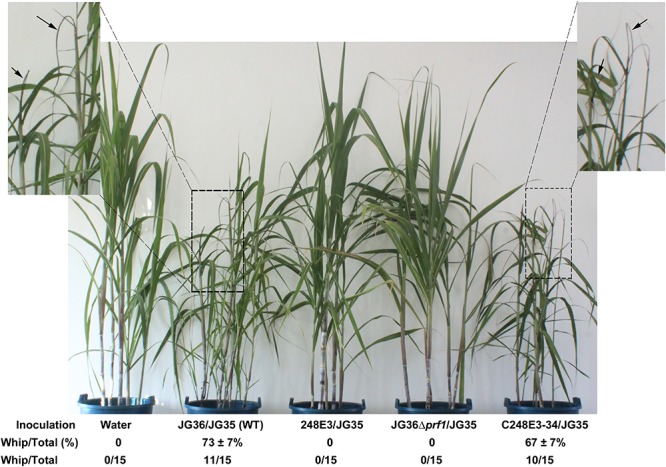
Pathogenicity assay of *SsPRF1* mutants. For pathogenicity assays, seedlings of the sugarcane variety ROC22 (highly susceptible to *S. scitamineum*) were inoculated by injecting the stem near the growing point with the mixed fungal cells of various pairwise combinations. Symptoms were documented and photographed at about 120 days post-inoculation. Dash-line boxed regions are enlarged for a better view of whip symptoms, denoted by arrows. Percentage of seedling showing whip formation out of total inoculated seedling was presented as Mean ± SE, derived from three independent repeats, each of which contained 5 seedlings. Number of whip/total seedlings was also indicated.

To determine if *SsPRF1* is involved in pathogenicity, we inoculated the sugarcane seedlings of variety ROC22 (highly susceptible to *S. scitamineum*) with the mixed sporidial cells of *S. scitamineum* in combinations of 248E3(*MAT-1*)/JG35(*MAT-2*), C248E3-34(*MAT-1*)/JG35(*MAT-2*), and JG36Δ*prf1*(*MAT-1*)/JG35(*MAT-2*), with wild-type strains JG35(*MAT-2*)/JG36(*MAT-1*) as positive control. It was found that sugarcane seedlings inoculated with either the T-DNA disrupted *SsPRF1* mutant (248E3) or the *SsPRF1* null mutant (JG36Δ*prf1*) remained healthy throughout the observed period of 120 days. In contrast, plants infected with wild-type combination JG36/JG35 displayed severe stunting, spindly stalks and upright and narrower leaves after 60 days, and whips emerged from the shoots at 90–120 days post-inoculation (Figure 4). In agreement with the restoration of mating, the complementation strain C248E3-34 incited the characteristic symptoms of “smut whip” when mixed with wild-type strain JG35 (*MAT-2*), comparable to those by the wild-type strains (Figure 4; *p* > 0.05).

We further quantified the relative fungal biomass in infected sugarcane stem tissue, at 3 days post-infection (dpi), and our result showed that the relative fungal biomass in the sugarcane seedlings infected by JG36Δ*prf1*(*MAT-1*)/JG35(*MAT-2*) was 139.65 ± 72.12% of that in the ones infected by the wild-type JG35(*MAT-2*)/JG36(*MAT-1*) mixture. Similarly, the relative fungal biomass from the 248E3(*MAT-1*)/JG35(*MAT-2*) infected seedlings was 118.21 ± 24.21% of that of WT. Difference between WT infection and mutant infection was not significant (*p* > 0.05). This result indicates that colonization of the plant tissue by WT or mutant mixed spordia at early stage were comparable, however, we could not differentiate the *in planta* fungal biomass detected by this assay was in sporidial or hyphal form. This result at least rules out the possibility that failure of developing disease symptom in the mutant infected sugarcane seedlings was not due to insufficient inoculum at the beginning.

### *SsPrf1* Regulates the Expression of Pheromone-Responsive Genes

Recently, the whole genome sequences of *S. scitamineum* strains from China, Brazil, Australia and South Africa have been reported (Que et al., 2014; Taniguti et al., 2015; Dutheil et al., 2016). The presence of pheromone receptor gene *SsPRA1* was first reported by Que et al. (2014) and the whole coding region was identified by RT-PCR and located to the *a1* locus (Yan et al., 2016b). During our BLAST search against *S. scitamineum* genome, the pheromone precursor gene *SsMFA1* with high identity to the pheromone precursor gene *mfa1.2* in *S. reilianum* was also identified and verified by RACE in the *a1* locus of *S. scitamineum* (GenBank: CP010914.1, 857085-857204).

**FIGURE 5:**
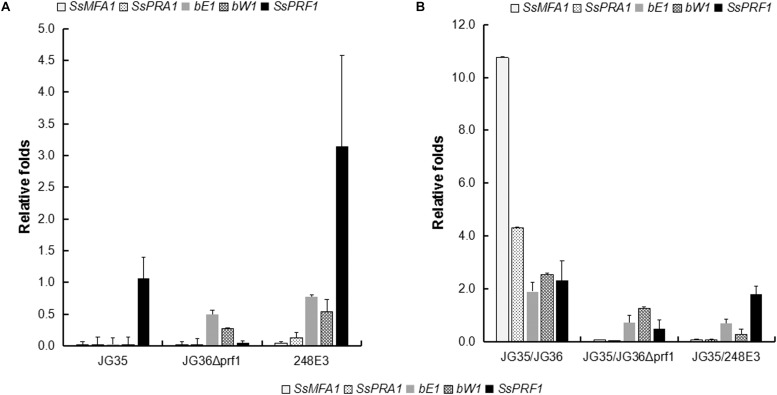
qRT-PCR analysis for expression of pheromone-inducible genes in *SsPRF1* mutants. The expression of *SsMFA1*, *SsPRA1*, *bE, bW*, and *SsPRF1* was analyzed in haploid strains JG35, JG36, JG36Δ*prf1*, and 248E3, cultivated alone **(A)** or together with JG35 sporidia **(B)**. Total RNA was isolated from haploid strains and mixtures of strains incubated for 2 days at 28°C on a rotary shaker. The gene-specific primer pairs 248E3-qF/248E3-qR, mfa1-qF/mfa1-qR, pra1-qF/pra1-qR, bE1-qF/bE1-qR, bW1-qF/bW1-qR, and 18S-qF/18S-qR (Table 1) were used to amplify the respective target genes. The *S. scitamineum 18S rRNA* gene was used as an endogenous control, and wild-type strain JG36 as the calibrator. The error bars represent standard deviations, derived from three biological repeats, each with three technical repeats.

To further dissect the function of *SsPRF1* in pheromone signaling and pathogenic development, transcription of the JG36-specific genes *SsMFA1* and *SsPRA1*, as well as the gene *SsPRF1* that is present in both JG35 and JG36, were assessed in the haploid strains of JG35, JG36, JG36Δ*prf1*, and 248E3, with or without pheromone induction from the opposite mating type strain. As shown in Figure 5A, *SsPRF1* was detected at comparable levels in JG36 and JG35, while undetectable in JG36Δ*prf1*, as expected. *SsMFA1* and *SsPRA1* expression was undetectable in JG35 (*MAT-2*) and was barely detectable in JG36Δ*prf1* or 248E3 (Figure 5A). However, it was interesting that the *SsPRF1* transcript level was threefold up-regulated in 248E3, suggesting that (1) the truncated *SsPRF1* was transcriptionally active, and (2) the mutated *SsPRF1* showed enhanced mRNA transcription.

To investigate the induction effect of the opposite mating type strain on mating type-related gene expression, JG36, JG36Δ*prf1*, and 248E3 were co-cultured with their compatible wild-type mating partner JG35 (*MAT-2*), which was assumed to provide *a2* pheromone and should stimulate the transcription of pheromone-inducible genes. As shown in Figure 5B, *SsMFA1* expression was significantly elevated (10.7-fold) in JG35/JG36 combination but not in JG35/JG36Δ*prf1* or JG35/248E3 combinations. *SsPRA1* expression was up-regulated by 4.3-fold in the JG35/JG36 combination but no increase in JG35/JG36Δ*prf1* or JG35/248E3 combinations could be detected, suggesting that *SsPRF1* in JG35 could not complement the *SsPRF1* defect in JG36Δ*prf1* and 248E3. It was interesting that *SsPRF1* expression was stimulated in JG35/JG36 combination (2.4-fold up-regulated) and in JG35/248E3 (1.9-fold up-regulated), implying that *SsPRF1* expression is regulated by signal(s) released during mating by the opposite mating type strains. In contrast, no stimulation in *SsPRF1* transcription was seen in JG35/JG36Δ*prf1*, whose transcript level reached about 50% of the wild-type level for *SsPRF1* mRNA. This reduced transcript level may likely be caused by the dilution of the *SsPRF1*-transcribing JG35 with *SsPRF1*-non-transcribing JG36Δ*prf1*.

We also assessed transcriptional regulation of the b locus genes *bE* and *bW* by qRT-PCR. Our result showed that transcription of *bE* or *bW* genes was slightly reduced in the sporidia of *SsPRF1* disruption or deletion mutants (Figure 5A), but the difference was not significant (*p* > 0.05). Under pheromone-induction condition (JG35/JG36), the b locus genes were upregulated (bE1: 1.90-folds; bW1: 2.54-folds; *p* < 0.05) while loss or disruption of *SsPRF1* in JG36 background made it unable to induce b locus gene transcription when mixed with wild-type JG35 sporidia (Figure 5B; *p* < 0.05). Overall, we conclude that SsPrf1 is responsible for transcriptional induction of a and b locus genes under mating condition.

### *a* Locus Genes Are Essential for *S. scitamineum* Mating/Filamentation

To further confirm that *S. scitamineum a*1 locus genes are functional in mating/filamentation, we next deleted *SsMFA1* and *SsPRA1* respectively, in JG36 (*MAT-1*) background. PCR amplification of the gene of interest (*SsMFA1* or *SsPRA1*) or the flanking sequences, using the primer pairs as indicated in Supplementary Figure S3A and listed in Table 1, confirmed successful gene deletion (Supplementary Figure S3B). We also performed Southern blot analysis to confirm deletion of *SsPRA1* gene (Supplementary Figure S3C).

**FIGURE 6:**
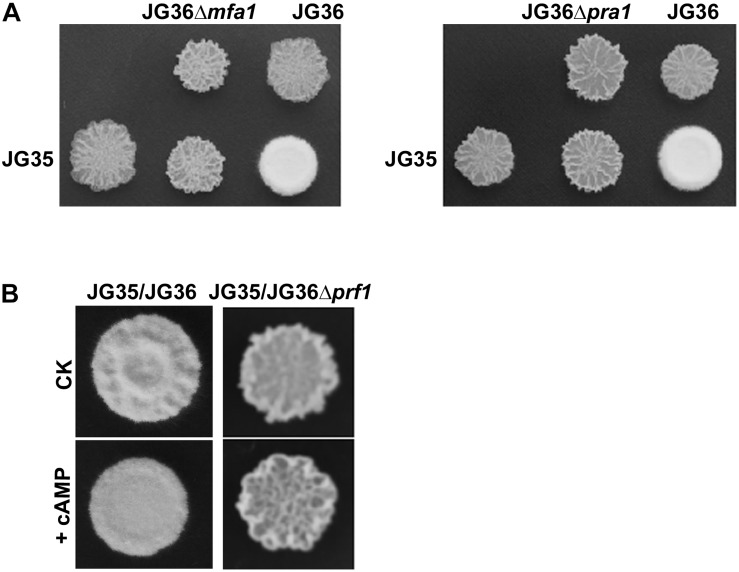
Characterization of the *a*1 locus genes *SsMFA1* and *SsPRA1* in *S. scitamineum* mating/filamentation. **(A)** Mating assays of mutants. Cultures of single wild-type strains and deletion mutants indicated on the top and on the left side of the photographs were spotted alone or in combination on YePS plates and incubated at 28şC for 3 days. Colonies on the first line on the photographs are wild-type strains or mutants spotted alone, while colonies on the second line are wild-type strains JG35 spotted alone or in combinations with strains indicated at the same columns. Positive mating reaction is indicated by the white fluffy colony morphology. Combinations of mutant strains show that mutants JG36Δ*mfa1* or JG36Δ*pra1* failed to form white and fluffy colonies when tested with compatible, wild-type mating partners. **(B)** Mating assay with addition of cAMP. JG36 (*MAT-1*) strain and *SsPRF1* gene deletion mutant were allowed to grown till O.D.600 = 1.0, and then mixed with equal volume of the JG35 (*MAT-2*) sporidia on solid medium. cAMP was mixed in the solid medium to reach the final concentration of 10 mM. Images were taken at 72 h post-inoculation.

Sporidia of the wild-type JG36 (*MAT-1*), JG36Δ*mfa1*, or JG36Δ*pra1* were respectively mixed and co-spotted with a compatible wild-type mating partner JG35 (*MAT-2*), to test their ability of mating/filamentation. We found that similar to the sexual mating between the Δ*prf1* mutant and wild-type mating-type strain, deletion of *SsMFA1* or *SsPRA1* led to failure of filamentation (Figure 6A). This confirms that *S. scitamineum a* locus gene *SsMFA1* and *SsPRA1* are functional in regulating fungal dimorphic switch, and likely acting at downstream of SsPrf1. This result is also consistent with a recent report on *SsMFA1* characterization (Sun et al., 2019).

### *SsPRF1* Is Independent/Downstream of cAMP-PKA Signaling Mediated ROS Regulation

Chang et al. (2018) recently reported a cAMP-PKA signaling pathway regulating *S. scitamineum* intracellular redox homeostasis, to promote mating/filamentation and host infection. As in the corn smut fungus *U. maydis*, Prf1 was shown to act at downstream of both PKA and MAPK pathway and not involved in redox regulation (Kaffarnik et al., 2003), we here assessed the epistatic interaction between SsPrf1 and the cAMP-PKA pathway, and the role (if any) of SsPrf1 in redox regulation.

As the mating/filamentation defect of the cAMP-PKA mutants could be fully or partially restored by exogenous addition of cAMP (Chang et al., 2018), we first tested the effect of cAMP on Δ*prf1* mutant. The Δ*prf1* mutant remained un-mating or un-filamentous when mixed with the compatible wild-type JG35 (*MAT-2*) sporidia (Figure 6B). This indicates that SsPrf1 may act at downstream of cAMP-PKA signaling pathway, as consistent with what has been reported in *U. maydis*. Alternatively, SsPrf1 may act in parallel with cAMP-PKA signaling.

We also tested tolerance to the oxidative stress caused by 1 mM H_2_O_2_, in comparison between the wild-type and the Δ*prf1* sporidia, and found no obvious difference (Supplementary Figure S4A). We next measured the intracellular H_2_O_2_ of the Δ*prf1* sporidia, and found that it was comparable to that of the wild-type sporidia (Supplementary Figure S4B; *p* > 0.05). Finally, exogenous addition of low concentration (0.1 mM) of H_2_O_2_ could not restore the mating/filamentation of the Δ*prf1* mixed with the wild-type sporidia (Supplementary Figure S4C), as it does to the cAMP-PKA mutants (Chang et al., 2018). These results suggest that SsPrf1 may not be involved in redox regulation in *S. scitamineum*, which is at downstream of cAMP-PKA pathway. Therefore the role of SsPrf1 in *S. scitamineum* mating/filamentation is more likely due to transcriptional regulation of the mating-type genes.

## Discussion

The morphological switch from yeast-like growth to filamentous growth occurs during the life style switch from the saprophytic to the biotrophic stage, and is critical for virulence of several animal- and plant-pathogenic fungi (Hartmann et al., 1996; Urban et al., 1996; Nadal et al., 2008; Elías-Villalobos et al., 2015). The human fungal pathogen *Candida albicans* can switch from a unicellular yeast form into pseudohyphae or hyphae, and such transition is important for virulence (Lo et al., 1997; Wartenberg et al., 2014). In the fungal plant pathogen *U. maydis*, morphological switching from yeast-like sporidia to dikaryotic hyphae occurs after sexual mating between two cells of opposite mating-types, a process under regulation of biallelic *a* and multiallelic *b* loci (Bölker et al., 1992; Gillissen et al., 1992; Spellig et al., 1994). *Sporisorium reilianum*, a smut fungus closely related to *U. maydis*, possesses three *a* alleles containing two active pheromone genes each, and at least five alleles for the *b* locus, that govern its ability of sexual mating and dimorphic switching essential for virulence (Schirawski et al., 2005). *S. scitamineum* is also a dimorphic pathogen with two different life styles, a saprophytic stage growing by budding as unicellular sporidia, and a pathogenic stage growing as dikaryotic hyphae. The morphological switch in *S. scitamineum* also depends on sexual mating (Que et al., 2014), but its regulatory mechanism is still not fully understood. The conserved *b* locus genes have also been functionally investigated, with the gene sequence encoding the pheromone receptors, *PRA1* and *PRA2*, annotated (Yan et al., 2016a, b). The *MFA1* and *MFA2* gene, respectively encoding the pheromone precursors in *MAT-1* and *MAT-2* mating-type strain, have both been annotated and characterized by reverse genetics (Lu et al., 2017; Sun et al., 2019). However, the pheromone response factor Prf1 that governs pheromone-induced transcription of *a* and *b* loci in *U. maydis*, has not yet been identified or characterized in *S. scitamineum*. Neither was it characterized in the biological function of the pheromone receptor encoding genes, *SsPRA1* and *SsPRA2*. As reported in this study, we identified a Prf1 ortholog in *S. scitamineum* and found that it was responsible for both basal and pheromone-induced expression of mating type genes of the *a1* locus. We further showed that deletion of the *a* locus gene *SsMFA1* or *SsPRA1* resulted in similar phenotype (Figure 6A) as the T-DNA insertion mutation or deletion mutant of the *SsPRF1* gene (Figure 3A), as well as the reported *MFA1* deletion phenotype (Sun et al., 2019). Our results provide new insight into the mechanism of pathogenicity of this important sugarcane pathogen, by confirming the biological function of the *a*1 locus gene in *S. scitamineum* mating/filamentation, and indicating that SsPrf1 plays a key role in pheromone signaling and filamentous growth in *S. scitamineum* through transcriptional induction of the mating locus.

Under pheromone-induced conditions, expression of *SsPRF1* in JG35/JG36 culture was about 2.4-fold up-regulated compared to that in the sporidial growth stage of the wild-type strains (Figure 5), suggesting that *SsPRF1* expression is up-regulated by mating. In this regard, it has been reported that in *U. maydis Prf1* expression was significantly induced by sexual pheromones (Hartmann et al., 1996). Since *SsPRF1* is present in both JG35 and JG36, we could not tell which strain may contribute to the increase in expression of the gene. By taking the expression levels in JG35/JG36Δ*prf1* and JG35/284E3 into account, we concluded that truncated *SsPRF1* in 284E3 was not functional, since the transcript level of *SsPRF1* in JG35/284E3 was at about the average of JG35 and 284E3 (Figure 5). Studies in *U. maydis* have shown that Prf1 undergoes post-translational modification (phosphorylation) for activation (Kaffarnik et al., 2003). Thus it would be of interest to investigate the upstream signal transduction pathways that switch on pheromone-stimulated gene expression through SsPrf1 phosphorylation.

Chang et al. (2018) reported that cAMP-PKA signal pathway regulates *S. scitamineum* mating/filamentation likely through regulation of intracellular redox homeostasis. We found that exougenous addition of cAMP could not restore mating/filamentation of the Δ*prf1* mutant, as it does to the cAMP-PKA mutants (Chang et al., 2018), indicating that SsPrf1 acts at downstream of cAMP-PKA signaling pathway. This is consistent with what has been reported in *U. maydis* (Kaffarnik et al., 2003). We found no obvious difference between the wild-type and the Δ*prf1* mutant in aspects of oxidative stress tolerance or intracellular H_2_O_2_ level (Supplementary Figures S4A,B). Also, addition of low concentration (0.1 mM) of H_2_O_2_ could not promote mating/filamentation in the Δ*prf1* mutant (under mating condition with the opposite mating-type sporidia, Supplementary Figure S4C), as it does to the wild-type strain or the cAMP-PKA mutants (Chang et al., 2018). These results confirmed that the SsPrf1 function is not relevant to intracellular redox homeostasis but may be solely on regulation of mating locus genes.

Overall, our present study, together with previous published functional study of *a* and *b* locus, completes the regulation network of *S. scitamineum* mating/filamentation at downstream of the cAMP-PKA signaling pathway, and in parallel of redox signaling.

## Data Availability

All datasets generated for this study are included in the manuscript and/or the Supplementary Files.

## Author Contributions

GZ, YD, EC, MY, GC, ZW, CZ, and BZ performed the experiments. PX, BC, CC, and ZJ conceived and designed the experiments. GZ, YD, CC, BC, and ZJ analyzed the data. GZ, BC, and ZJ contributed reagents, materials, and analysis tools. GZ and YD wrote the manuscript. All authors read and approved the final manuscript.

## Conflict of Interest Statement

The authors declare that the research was conducted in the absence of any commercial or financial relationships that could be construed as a potential conflict of interest.
