# Supplementary material for: Identification and Functional Analysis of the Pheromone Response Factor Gene of Sporisorium scitamineum
Source: Front Microbiol. 2019 Sep 10;10:2115. doi: 10.3389/fmicb.2019.02115 (PMC6747018; doi:10.3389/fmicb.2019.02115)
Supplement: Supplementary file 1 [file Data_Sheet_1.PDF]

Supplementary Information

Identification and Functional Analysis of the Pheromone Response Factor Gene of *Sporisorium scitamineum*

Guining Zhu, Yizhen Deng, Enping Cai, Meixin Yan, Guobing Cui, Zhiqiang Wang, Chengwu Zou, Bin Zhang, Pinggen Xi, Changqing Chang, Baoshan Chen[1], Zide Jiang[1]

1. To whom correspondence should be addressed. Email: chenyaoj@gxu.edu.cn (B Chen), zdjiang@scau.edu.cn (Z Jiang).

This PDF file includes

Supplementary Figures and Legends

Figure S1

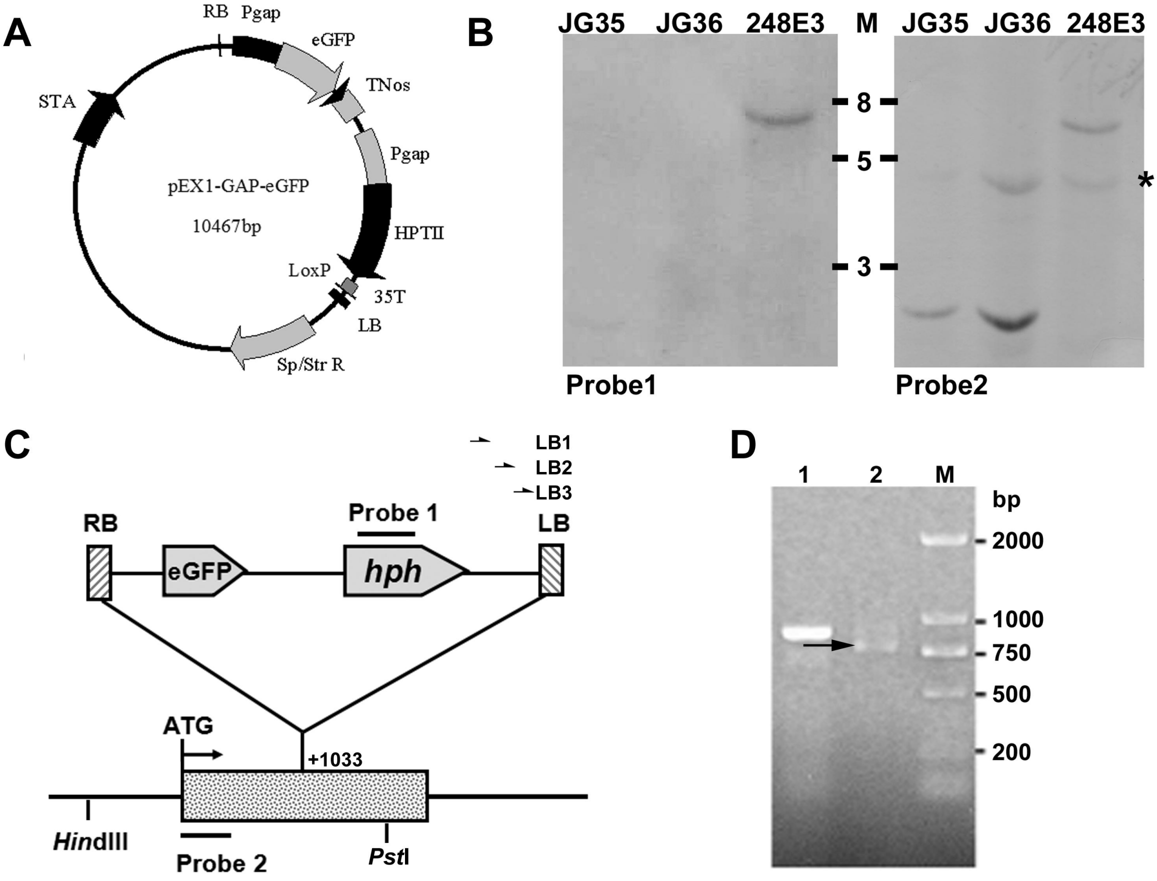

Figure S1. Identification of *S. scitamineum PRF1* ortholog. A, schematic diagram of the plasmid pEX1-GAP-eGFP used for generating the T-DNA insertion mutant library. LB and RB are the left and right border sequences of T-DNA, respectively. Pgap, *GAP* promoter; eGFP, enhanced GFP; TNos, Nos terminator; HP TII, *hph* ORF; 35T, 35S terminator; Sp/Str R, streptomycin resistance; STA, replication origin region from pVS1 plasmid. **B**, Southern blot analysis of the wild-type strains JG35, JG36, and the T-DNA insertion mutant 248E3. Total genomic DNA was digested with *Hin*dIII+*Pst*I and probed with *hph* fragment (probe 1), or *SsPRF1* specific fragment (probe 2) as shown in Figure 2C. Molecular markers are indicated on the left. With Probe 1 the wild-type strains had no band detected, while 248E3 showed a single

band of approximate 6.7 kb. With Probe 2, the wild-type strains showed a 2.7 kb band expected as the *SsPRF1* fragment between *Hin*dIII and *Pst*I enzyme sites, and 248E3 of 6.7 kb, with T-DNA insertion within *SsPRF1* fragment. Asterisk (*) denotes non-specific detection. **C**, schematic diagram showing T-DNA insertion site of mutant 248E3. Probe 1: *hph* resistance gene. Probe 2: the foreside of *SSCI14340.1*. LB and RB are the left and right border sequences of T-DNA respectively. This diagram was not drawn to scale. **D**, electrophoretic analysis of hiTAIL-PCR products. 1-2: The products of primary and secondary-amplification, respectively.

Figure S2

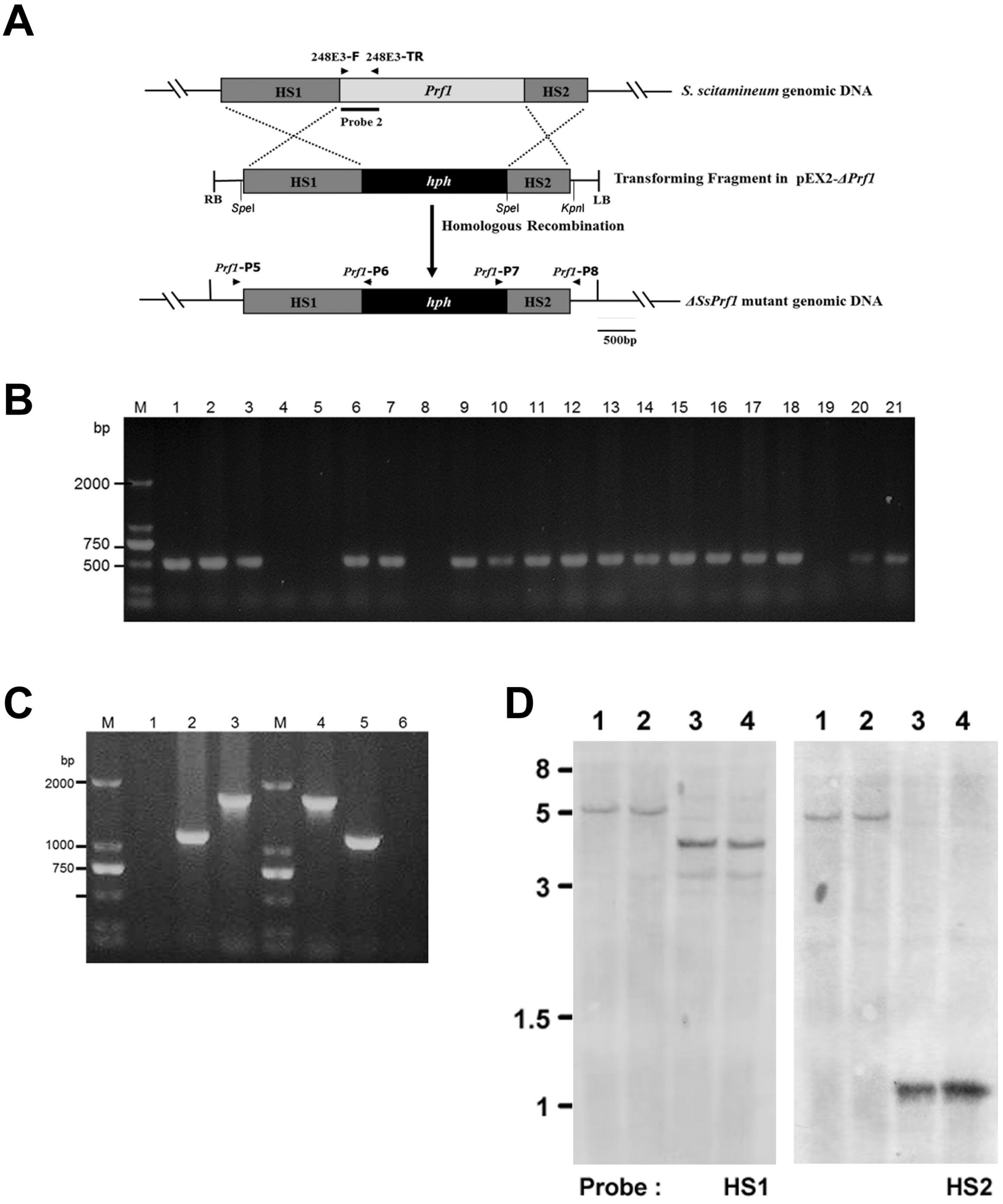

Figure S2. Generation of *SsPRF1* deletion mutants. A, schematic diagram for *SsPRF1* deletion using homologous recombination strategy. **B**, transformants selected by PCR. Amplification of a genomic fragment (510 bp) specific to the *SsPRF1* gene, using the specific primer pair 248E3-F/248E3-TR (Table 1). Four *SsPRF1* null mutants (4, 5, 8 and 19) produced no *SsPRF1*-specific band, indicative as successful deletion of the target gene. **C**, PCR amplification to verify *SsPRF1* null mutants. M:

DL2000 DNA ladders; 1, 2, 3: JG35*Δprf1* amplified by 248E3-F/248E3-TR, *prf1*-P7/*prf1*-P8 and *prf1*-P5/*prf1*-P6, respectively; 4, 5, 6: JG36*Δprf1* amplified by *prf1*-P5/*prf1*-P6, *prf1*-P7/*prf1*-P8 and 248E3-F/248E3-TR, respectively. Position of the primers are indicated in A. **D**, Southern blotting confirmed deletion of *SsPRF1*. 1, 2, 3, 4: JG35, JG36, JG35*Δprf1*, JG36*Δprf1*. Molecular size markers in kilobasepair units (kb) are indicated on the left. DNA digested with *SpeI+KpnI*, and probed respectively with HS1 or HS2 fragment as shown in A. Detected bands for wild-type strains are expected to be 5.0 kb (both HS1 and HS2 probes), and deletion mutants 3.5 kb (probed by HS1) or 1.0 kb (probed by HS2).

Figure S3

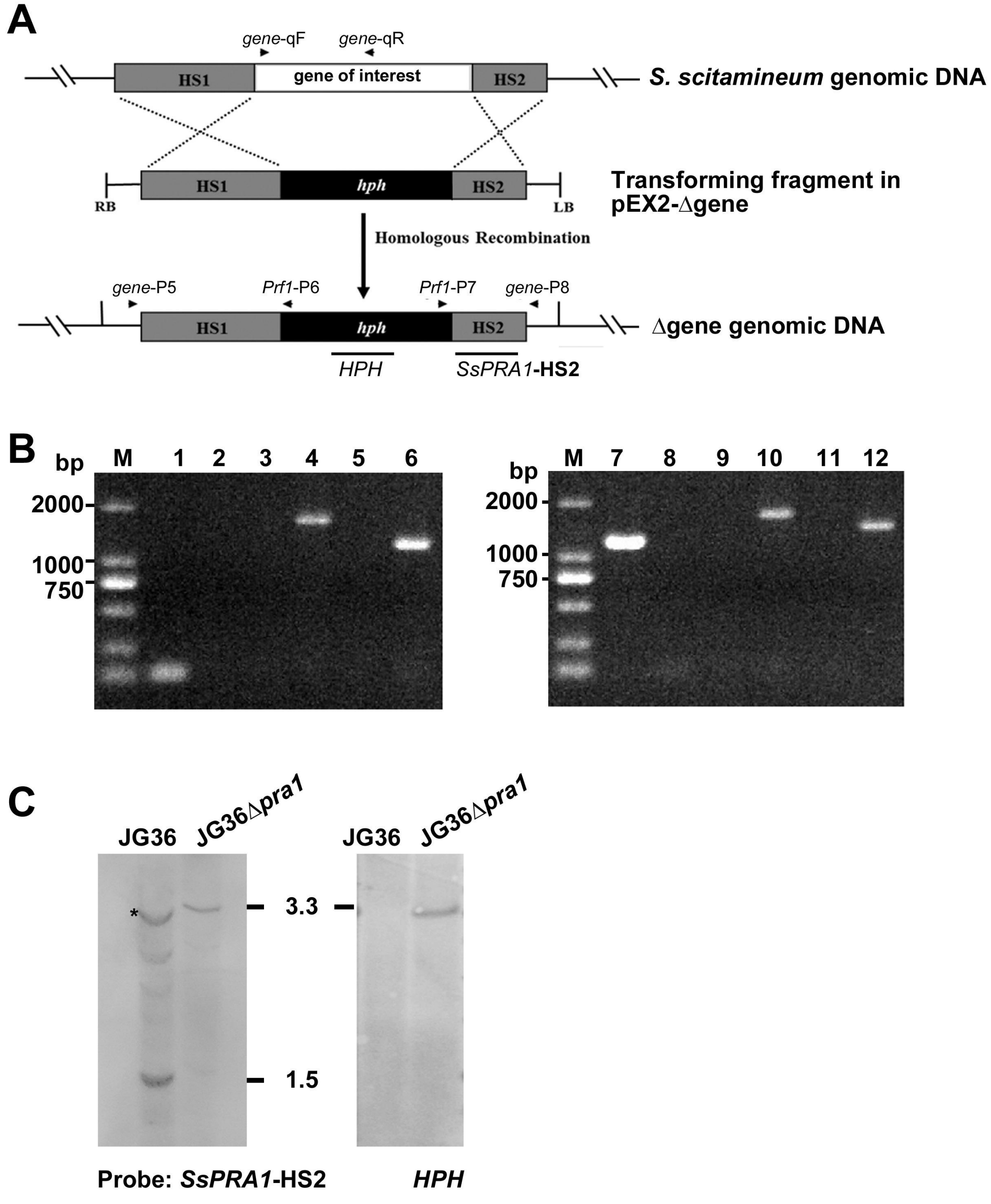

Figure S3. Generation of *a*1 locus gene deletion mutants. A, schematic diagram (not drawn to scale) for *SsMFA1* or *SsPRA1* deletion using homologous recombination strategy. Gene of interest denotes to *SsMFA1* or *SsPRA1*. *gene*-F/*gene*-R primers denote to *mfa1*-qF/*mfa1*-qR, or *pra1*-F/*pra1*-R primer pairs used in **B**. *gene*-P5 or *gene*-P8 primer was denoted to *mfa1*-P5, *pra1*-P5 or *mfa1*-P8, *pra1*-P8, repectively

paired with *prf1*-P6 or *prf1*-P7, to amplify the flanking sequence and confirm gene deletion as shown in **B**. The DNA sequences of the primers were listed in Table 1. **B**, PCR amplification to verify *MFA1* or *PRA1* null mutants. M: DL2000 DNA ladders; 1, 3, 5, 7, 9, 11: JG36 amplified by *mfa1*-qF/*mfa1*-qR, *mfa1*-P5/*prf1*-P6, *prf1*-P7/*mfa1*-P8, *pra1*-F/*pra1*-R, *pra1*-P5/*prf1*-P6 and *prf1*-P7/*pra1*-P8, respectively; 2, 4, 6: JG36*Δmfa1* amplified by *mfa1*-qF/*mfa1*-qR, *mfa1*-P5/*prf1*-P6 and *prf1*-P7/*mfa1*-P8, respectively; 8, 10, 12: JG36*Δpra1* amplified by *pra1*-F/*pra1*-R, *pra1*-P5/*prf1*-P6 and *prf1*-P7/*pra1*-P8, respectively. Position of the primers are indicated in **A**. **C,** Southern blotting confirmed deletion of *SsPRA1*. Genomic DNA of JG36 and JG36*Δpra1* was digested with *Hind*III+*Pst*I, and probed respectively with HS2 fragment of *SsPRA1* or *HPH* fragment as shown in A. Molecular size markers in kilobasepair units (kb) are indicated. Detected bands for wild-type strains are expected to be 1.5 kb for *SsPRA1*-HS2 probes, and deletion mutants 3.3 kb (probed by *SsPRA1*-HS2 or *HPH*).

Figure S4

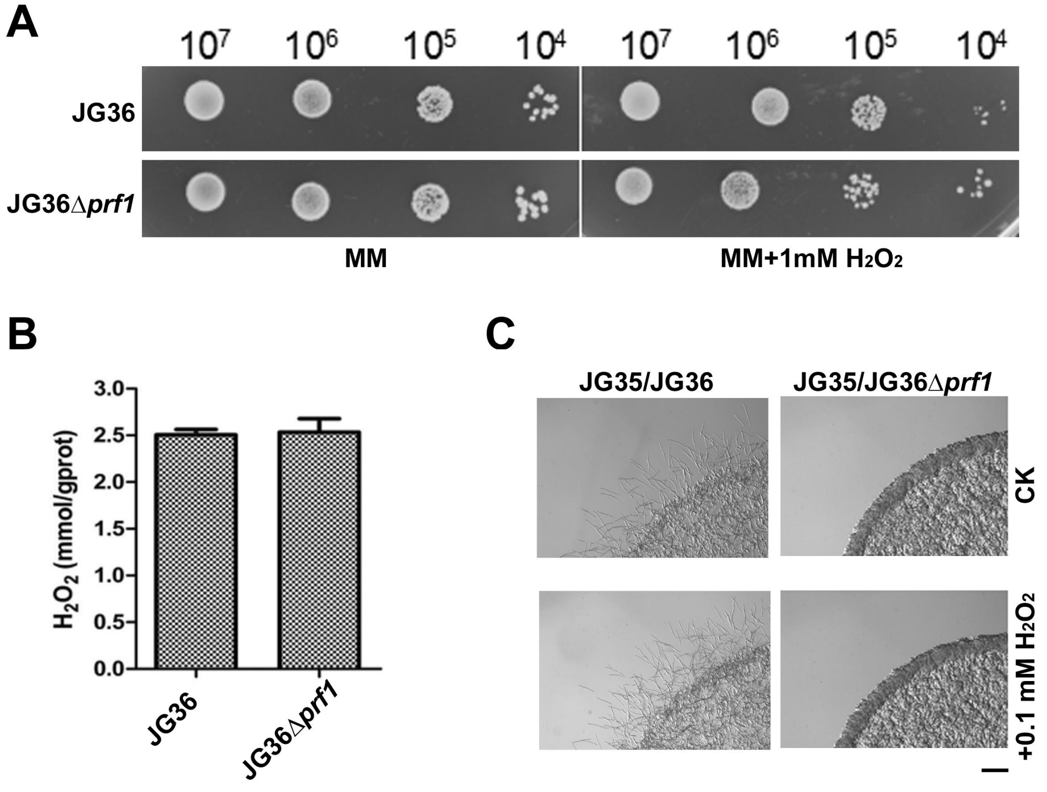

Figure S4. Tolerance to oxidative stress and measurement of intracellular H_2O_2 in the *ssprf1*Δ mutant. A, sporidia from JG36 (*MAT-1*) strain and *SsPRF1* gene deletion mutant, of serial dilution as denoted on the top of the images, were spotted on solid MM medium, with or without supplement of 1 mM H_2O_2. Images were taken at 72 h post inoculation. **B,** measurement of intracellular H_2O_2 in the JG36 (*MAT-1*) strain and *SsPRF1* gene deletion mutant. Intracellular H_2O_2 was visualized by DAB staining and quantified by spectrophotometer measurement at the wavelength of 405 nm, with its reaction product with molybdic acid. The error bars represent standard deviations, derived from three biological repeats. **C,** sporidia from JG36 (*MAT-1*) strain or *SsPRF1* gene deletion mutant, were mixed with the JG35 (*MAT-2*) sporidia of equal volume and spotted to solid MM medium. 0.1 mM H_2O_2 was added to test

their effect on *S. scitamineum* mating/filamentation. Images were taken with Leica M165FC stered epifluorescence microscope equipped with MC170-HD camera at 12 h post inoculation. Scale bar = 0.2 mm.
